# Specific expression of MUC21 in micropapillary elements of lung adenocarcinomas – Implications for the progression of *EGFR*-mutated lung adenocarcinomas

**DOI:** 10.1371/journal.pone.0215237

**Published:** 2019-04-11

**Authors:** Mai Matsumura, Koji Okudela, Yu Nakashima, Hideaki Mitsui, Kaori Denda-Nagai, Takehisa Suzuki, Hiromasa Arai, Shigeaki Umeda, Yoko Tateishi, Chihiro Koike, Toshiaki Kataoka, Tatsuro Irimura, Kenichi Ohashi

**Affiliations:** 1 Department of Pathology, Yokohama City University, School of Medicine, Yokohama, Japan; 2 Division of Glycobiologics, Intractable Disease Research Center, Juntendo University Graduate School of Medicine, Tokyo, Japan; 3 Division of Surgery, Kanagawa Prefectural Cardiovascular and Respiratory Center Hospital, Yokohama, Japan; University of South Alabama Mitchell Cancer Institute, UNITED STATES

## Abstract

We investigated the significance of MUC21 in *EGFR*-mutated lung adenocarcinoma (LADC). Two-hundred forty-one surgically resected LADCs (116 *EGFR*-mutated and 125 wild-type tumors) were examined for immunohistochemical expression of MUC21 protein. A polyclonal antibody and two monoclonal antibodies (heM21C and heM21D) that bind differentially glycosylated MUC21 epitopes were used, and MUC21 proteins detected by these antibodies were named MUC21P, MUC21C, and MUC21D, respectively. MUC21 mRNA levels were semi-quantified and classified into “high” and “low”. Among the immunohistochemical expression detected by three different antibodies, high expressors tended to be related to *EGFR* mutations. The three varieties of the immunohistochemical expressions were related to different histological elements in the *EGFR*-mutated LADCs. Either MUC21P or MUC21C high expressors had a higher proportion of lepidic elements with low papillary structure and micropapillary elements. MUC21D high expressors had a significantly higher proportion of micropapillary elements (Mann-Whitney test P ≤0.0001). Furthermore, MUC21D high expressors showed high incidence of lymphatic canal invasion and lymph node metastasis (Pearson x2 test, *P* = 0.0021, *P* = 0.0125), and a significantly higher recurrence rate (5-year recurrence-free survival 50.7% vs. 73.8%, log-rank test *P* = 0.0495). MUC21 proteins with a specific glycosylation status may be involved in the progression of *EGFR*-mutated LADCs, particularly at the stage where tumors are transforming from pure lepidic to micropapillary through low papillary lepidic lesions.

## Introduction

*EGFR*-mutated lung adenocarcinoma (LADC) develops through a distinct histogenesis in which micropapillary (mPAP) elements promote tumor progression [1]. However, the molecular basis of mPAP elements is not fully understood.

Mucin family proteins help determine the pathobiological features of neoplastic cells. Some mucins, MUC1 (CA15-3) and MUC16 (CA125), are useful biomarkers to monitor malignant neoplasms. Our preliminary comprehensive immunohistochemical examination of mucin proteins, including MUC-1, 2, 3B, 4, 5AC, 5B, 6, 7, 9, and 21, in LADCs revealed that MUC21 was strongly expressed in mPAP elements. MUC21 was first identified as a human homologue of mouse Muc21/epiglycanin from human cervical carcinoma cell lines [2]. MUC21 is a transmembrane mucin and is expressed in various human neoplasms including lung carcinomas [2] [3] [4] and is linked with aggressive behavior of neoplastic cells [5].

Here, we examined immunohistochemical expressions using three different antibodies that bind to differentially glycosylated epitopes [3] and investigated the potential significance of MUC21 in *EGFR*-mutated LADCs in relationship to mPAP elements.

## Material and methods

### Patients

Two hundred and forty-one surgically resected LADCs were examined. These tumors were resected at the Kanagawa Prefectural Cardiovascular and Respiratory Center between January 1997 and December 2013. Informed consent for the use of these samples for research purposes was obtained in writing. The ethics committees of Kanagawa Prefectural Cardiovascular and Respiratory Center and Yokohama City University approved the research plan.

### Histopathological examination

Formalin-fixed paraffin-embedded tissue sections were cut and stained with hematoxylin and eosin. The proportions of histological elements (lepidic, acinar, papillary, mPAP, and solid elements) were described in 5% increments according to the World Health Organization (WHO) classification [6]. Lepidic elements were divided into lepidic elements with low papillary structure (LEPL) and pure lepidic elements as described previously [7]. Two pathologists (M.M. and K.O.) reviewed all hematoxylin and eosin-stained tissue sections and reached a consensus on the proportions of each histological element.

### Immunohistochemical examination

We used three antibodies that recognize different epitopes of MUC21. One polyclonal antibody (Novus Biologicals, Littleton, CO) was generated against the 20-amino-acid cytoplasmic tail, and two monoclonal antibodies (mAb heM21C and mAb heM21D) bind differentially to glycosylated MUC21. The mAb heM21C binds MUC21 modified by an N-acetylgalactosamine (Tn-MUC21), galactose glycosylation of Tn-MUC21 (T-MUC21), and sialic acid attached to T-MUC21 (sialyl T-MUC21), whereas the mAb heM21D binds to both the unmodified core polypeptide of MUC21 and Tn-MUC21 [3].

Immunohistochemistry using the specific antibodies against MUC21 was performed manually. Antigen retrieval was performed using autoclave treatment. Immunoreactivity was visualized with the Envision detection system (DAKO, Ely, UK). The reactions were developed using commercially available DAB, and counterstaining was performed with hematoxylin. The intensity of MUC21 in neoplastic cells was judged as negative (intensity 0), weak (intensity 1), or strong (intensity 2). We defined the MUC21 score as follows: [score = 1× (proportion of area with weak intensity [%, in 5% increments]) + 2× (proportion of area with strong intensity [%, in 5% increments])]. Two pathologists (M.M. and K.O.) reviewed all MUC21-stained tissue sections and reached a consensus on the intensity and proportions of the positive areas.

### Quantitative RT-PCR

MUC21 mRNA expression was examined in 22 tumors that had RNA quality sufficient for quantitative analysis. Total RNA was extracted using an RNAeasy kit (Qiagen, Venlo, Netherlands). First-strand cDNA was synthesized from total RNA using the SuperScript First-Strand Synthesis System according to the manufacturer’s instructions (Invitrogen, Carlsbad, CA). The cDNA was used as a template for real-time PCR with SYBR Premix EXTaq (Takara, Tokyo, Japan) and run on a Thermal Cycler DICE real time PCR machine. Primers used for the detection of MUC21 were forward (F) 5’-ggccagcgctctgacatgcagaa and reverse (R) 5’-ctggggacagagaacggtcctcc. Primers used for GAPDH were F 5’-ggtcgtattgggcgcctggt and R 5’-tactcagcgccagcatcgcc. The mean and standard deviation of the relative copy number of MUC21 to GAPDH mRNA were statistically obtained from triplicate reactions.

### Statistical analysis

Pearson’s chi-square test or Fisher’s exact test were used in combination with the Mann-Whitney test to analyze categorical and continuous variables, respectively. Recurrence curves were plotted using the Kaplan-Meier method. Differences in the recurrence-free survival (RFS) were analyzed using the log-rank test. *P* values <0.05 were considered significant. All analyses were performed using JMP 9.0.2 (SAS Institute, Cary, NC, USA).

## Results

### Scoring MUC21 immunohistochemical expression

**Fig 1:**
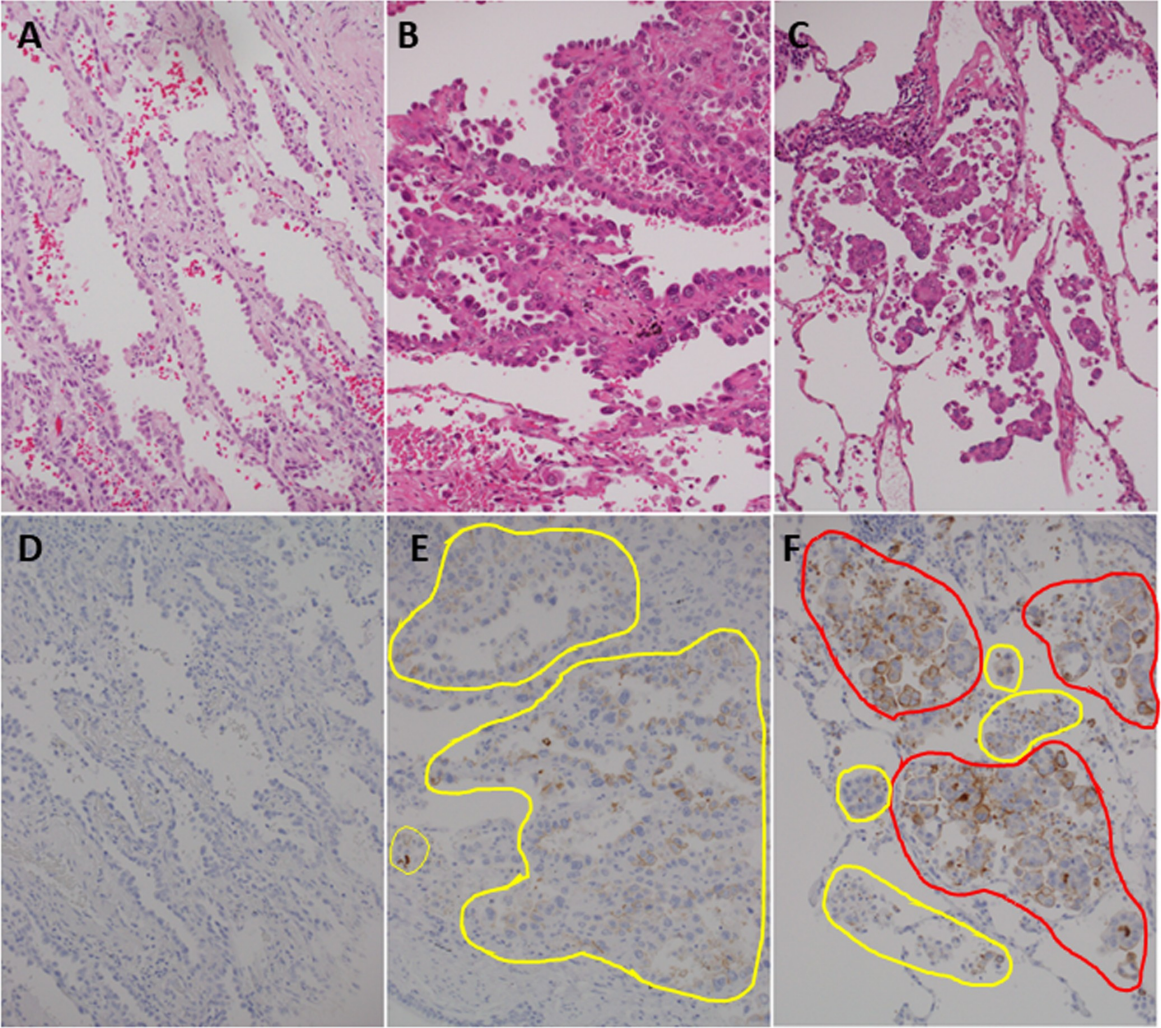
Representative images of tumors with varying immunohistochemical scores. Tumor cells with weak signal (intensity 1) are in the yellow circle, and tumor cells with strong signal (intensity 2) are in the red circle. In the left panels, positive signal is not detected throughout the tumor (Score 0) (A and D). In the center panels, weak signal is detected in most (90%) neoplastic cells (score 90 = 1×90) (B and E). In the right panels, strong signal is detected in most (80%) neoplastic cells (score 180 = 1×20 + 2×80) (C and F). A, B, and C are the hematoxylin and eosin stain. D, E, and F are immunohistochemistry for MUC21 with the polyclonal antibody. Magnification of all images is 200×.

Most adenocarcinomas expressed MUC21 (if tumors with very focal expression were included, a positive frequency, for example MUC21 expression detected by the polyclonal antibody, was 77.6% (187/241) of whole LADCs, and 87.1% (101/116) of *EGFR*-mutated LADCs). The level of MUC21 immunohistochemical expression varied among individual tumors and histological elements. The levels were semi-quantified by a scoring system described in the Materials and Methods. Representative images of tumors with different immunohistochemical scores are shown (Fig 1).

### Setting cut-off values to divide immunohistochemical scores into “high” and “low”

**Fig 2:**
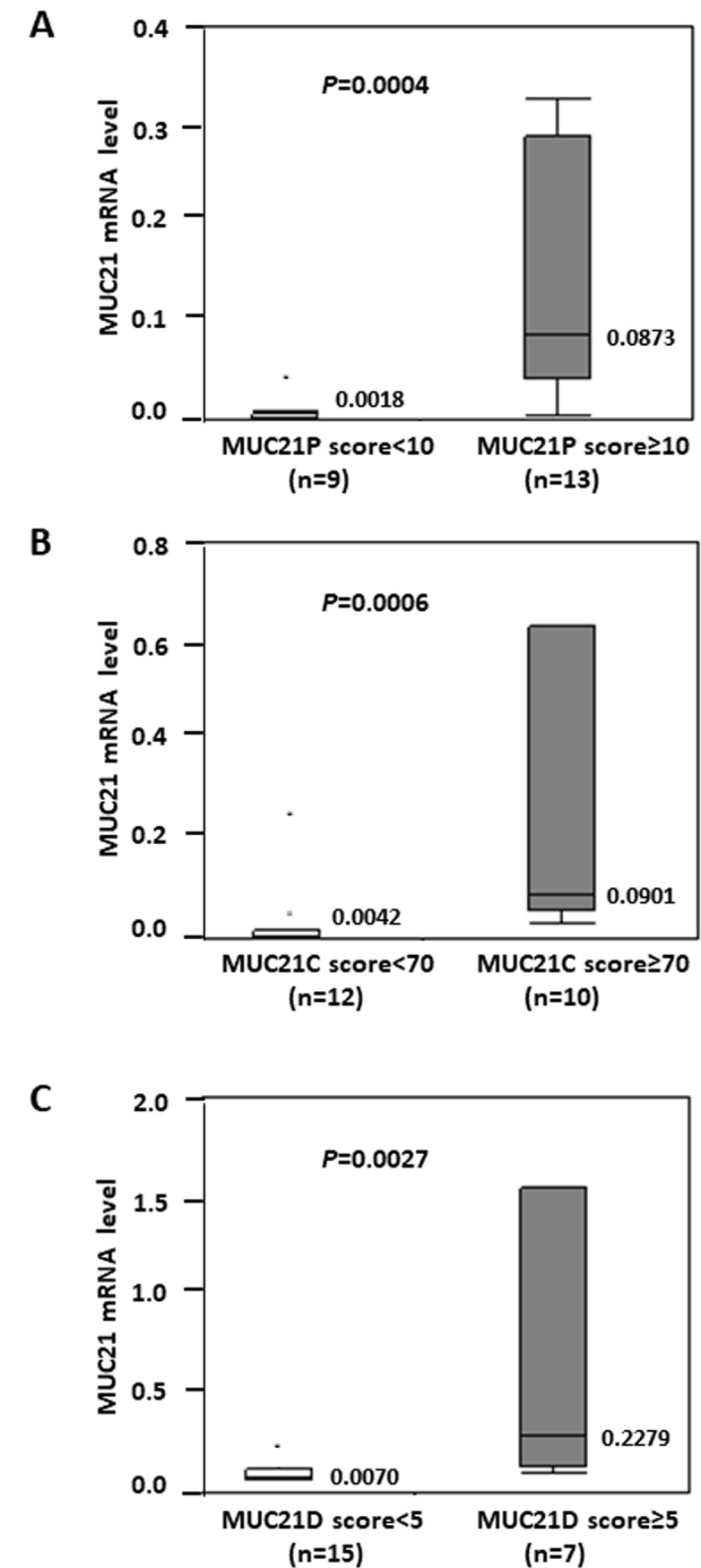
Correlations between MUC21 immunohistochemical scores and MUC21 mRNA levels. MUC21 mRNA levels are displayed as a box-and-whiskers plot (median, thick line; 25th to 75th percentile, box; 10th to 90th percentile, whiskers; outliers, dots). The cut-off immunohistochemical score was set to obtain the lowest *P*-value for the correlation between MUC21 mRNA and each MUC21 immunohistochemical expression; MUC21P (A, cut-off value of 10), MUC21C (B, cut-off value of 70), and MUC21D (C, cut-off value of 5). Median values are described in the graphs. *P* values were calculated using the Mann-Whitney test. n, number of tumors examined. A total of 22 *EGFR*-mutated lung adenocarcinomas were examined.

The association between the immunohistochemical score and MUC21 mRNA levels was analyzed using the Mann-Whitney test (Fig 2). MUC21 proteins were detected by three antibodies: polyclonal, mAb heM21C, and mAb heM21D named MUC21P, MUC21C, and MUC21D, respectively. A significant association with the lowest *P* values (MUC21P; *P* = 0.0004, MUC21C; *P* = 0.0006, MUC21D; *P* = 0.0027) was obtained when the immunohistochemical scores were divided into “high” and “low” by cut-off values of MUC21P = 10, MUC21C = 70, and MUC21D = 5. We used categories of “high expressors” and “low expressors” classified by these cut-off values for subsequent correlation analyses.

### Relationship between MUC21 immunohistochemical levels and the clinicopathological characteristics of LADCs

The clinicopathological factors of 241 LADCs are shown in Table 1. High expressors of MUC21, in particular MUC21P and MUC21C, were significantly related to female, non-smoker, terminal respiratory unit type, and *EGFR* mutations (Table 1). The results supported our expectation that MUC21 was preferentially involved in the progression of *EGFR*-mutated LADCs.

**Table 1:** Relationships between MUC21 expression and clinicopathological factors in lung adenocarcinomas.

	MUC21P low expressor (N = 114)	MUC21P high expressor (N = 127)		MUC21C low expressor (N = 164)	MUC21C high expressor (N = 77)		MUC21D low expressor (N = 191)	MUC21D high expressor (N = 50)	
	% (N)	% (N)	*P*	% (N)	% (N)	*P*	% (N)	% (N)	*P*
**Age**			0.8928			0.5657			0.622
Young(≤ 65)	36.0(41)	34.7(44)		36.6(60)	32.5(25)		36.1(69)	32.0(16)	
Old (> 65)	64.0(73)	65.4(83)		63.4(104)	67.5(52)		63.4(122)	68.0(34)	
Median(range)	68(36–85)	69(37–86)		68(36–86)	70(40–84)		68 (36–85)	72(37–86)	
**Sex**			0.0068*			0.0126*			0.5264
Female	42.1 (48)	59.8 (76)		45.7 (75)	63.6 (49)		50.3 (96)	56.0 (28)	
Male	57.9 (66)	40.2 (51)		54.3 (89)	36.4 (28)		49.7 (95)	44.0 (22)	
**Smoking status**			0.036*			0.0005*			0.6364
Never smoked	40.4 (46)	52.8 (67)		39.0 (64)	63.6 (49)		46.1 (88)	50.0 (25)	
Smoking	59.7 (68)	47.2 (60)		61.0(100)	36.4 (28)		53.9(103)	50.0 (25)	
**Histological subtype**			0.2711			0.1337			0.0148*
Lepidic**	60.0 (68)	52.0 (66)		53.7 (88)	59.7 (46)		61.3(117)	34.0 (17)	
Acinar	22.8 (26)	30.7 (39)		29.3 (48)	22.1 (17)		23.6 (45)	40.0 (20)	
Papillary	4.4 (5)	8.7 (11)		4.3 (7)	11.7 (9)		5.2 (10)	12.0 (6)	
Micropapillary	0.9 (1)	0.8 (1)		1.2 (2)	0.0 (0)		0.5 (1)	2.0 (1)	
Solid	10.5 (12)	7.9 (10)		10.4 (17)	6.5 (5)		8.4 (16)	12.0 (6)	
Mucinous	1.8 (2)	0 (0)		1.2 (2)	0 (0)		1.1 (2)	0 (0)	
**Cytological subtype**			0.0002*			0.009*			0.4159
TRU	77.2 (88)	94.5 (120)		81.7(134)	96.1 (74)		75.4 (89)	94.5(121)	
Non-TRU/BSE	14.0 (16)	1.6 (2)		10.4 (17)	1.3 (1)		16.1 (19)	1.6 (2)	
Unclassifiable	8.8 (10)	3.9 (5)		7.9 (13)	2.6 (2)		8.5 (10)	3.9 (5)	
***EGFR* mutation**			<0.0001*			0.0185*			0.1521
Mutation	32.5 (37)	62.2 (79)		42.7 (70)	59.7 (46)		45.6 (87)	58.0 (29)	
Wild type	67.5 (77)	37.8 (48)		57.3 (94)	40.3 (31)		54.5(104)	42.0 (21)	

High expressor was defined as tumor with a score more than or equal to 10 (MUC21P), 70 (MUC21C), 5 (MUC21D); Low expressor was defined as tumor with a score less than 10 (MUC21P), 70 (MUC21C), 5 (MUC21D); N, number; TRU, terminal respiratory unit; BSE, bronchial surface epithelium; *P* values were calculated using the Fisher’s exact test; Asterisk(*), statistically significant; **Lepidic histological subtypes in this analysis includes adenocarcinoma in situ and minimally invasive adenocarcinoma.

### Differential immunohistochemical expression of MUC21 in different histological elements among *EGFR*-mutated LADCs

**Fig 3:**
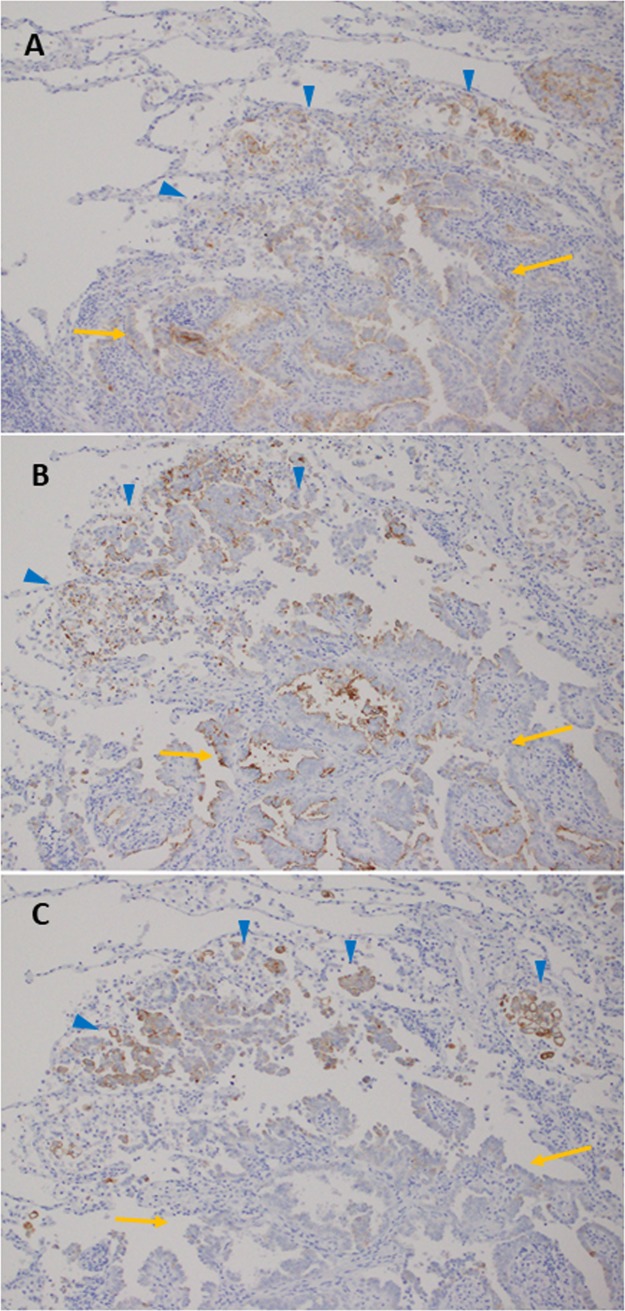
Representative images of three different varieties of MUC21 immunohistochemical expression. MUC21P (A) and MUC21C (B) were expressed in lepidic elements with low papillary structure (yellow arrow) and micropapillary (mPAP) elements (blue arrowhead). MUC21D (C) was expressed only in mPAP elements (blue arrowhead). Magnification of all images is 100×.

The immunohistochemical expression patterns of MUC21P and MUC21C were similar to each other, and both were preferentially expressed in mPAP and LEPL elements (Fig 3A and 3B). On the other hand, MUC21D expression was mostly specific to the mPAP element (positive signals were rarely found in LEPL elements) (Fig 3C).

**Fig 4:**
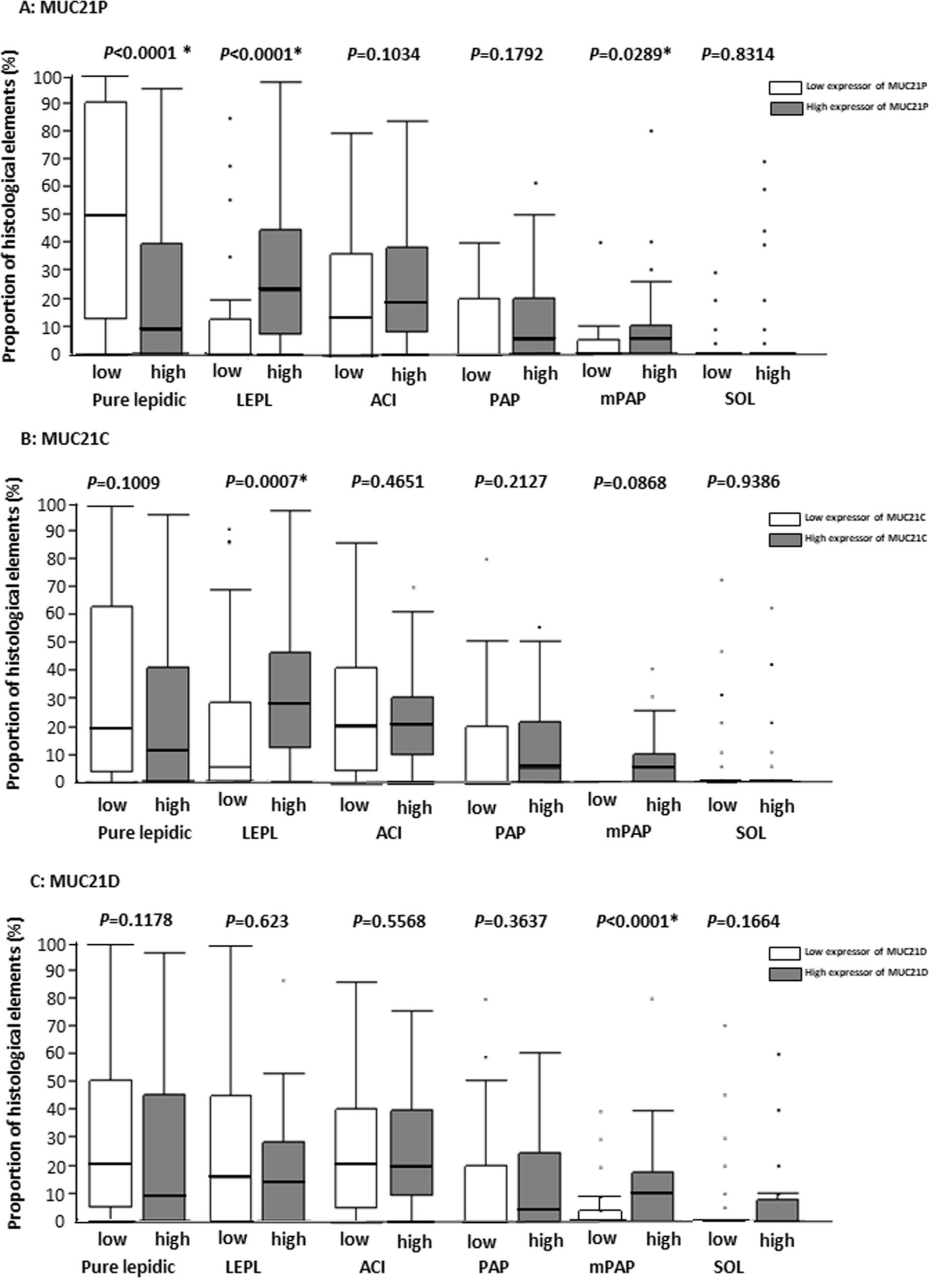
The correlations between the proportions of histological elements and the levels of MUC21 immunohistochemical expressions. Proportions of histological elements are displayed as a box-and-whiskers plot (median, thick line; 25th to 75th percentile, box; 10th to 90th percentile, whiskers; outliers, dots). A total of 116 *EGFR*-mutated lung adenocarcinomas were examined. *P*-values were calculated using the Mann-Whitney test. High expressors of MUC21P had a significantly higher proportion of lepidic elements with low papillary structure (LEPL) and micropapillary (mPAP) elements (A). High expressors of MUC21C had a significantly greater proportion of LEPL (B). High expressors of MUC21D had a significantly greater proportion of mPAP elements (C). Abbreviations, LEPL; lepidic element with low papillary structure, ACI, acinar; PAP, papillary; mPAP, micropapillary; SOL, solid; Asterisk (*), statistically significant.

To confirm these relationships, we measured the proportions of the histological elements (pure lepidic, LEPL, acinar, papillary, mPAP, and solid) in *EGFR*-mutated LADCs, and analyzed differences in the proportions between high and low expressors of MUC21P, MUC21C, and MUC21D (The minimal underlying data set are shown in S1 Table). High expressors of MUC21P had a significantly greater proportion of LEPL and mPAP elements (Fig 4A, Mann-Whitney test *P* <0.0289). High expressors of MUC21C had a significantly greater proportion of LEPL elements (Fig 4B, Mann-Whitney test *P* = 0.0007). High expressors of MUC21D had a significantly higher proportion of mPAP elements (Fig 4C, Mann-Whitney test *P* <0.0001).

Taken together, both MUC21P and MUC21C were preferentially expressed in either mPAP or LEPL elements, and MUC21D was more specific to mPAP elements.

### Relationship between MUC21 levels and highly malignant pathological factors in *EGFR*-mutated LADCs

**Fig 5:**
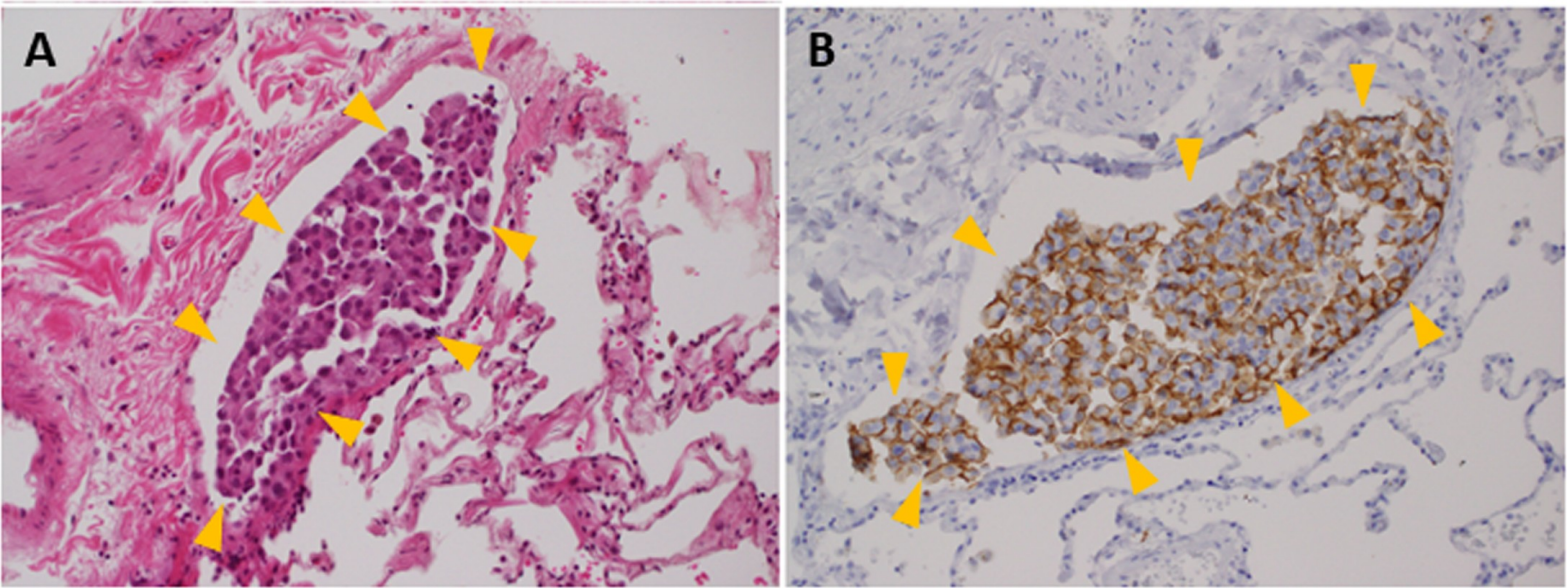
Representative images of lymphatic canal invasion in a MUC21D high expressor (A, hematoxylin and eosin stain; B, immunohistochemistry for MUC21D). A cluster of tumor cells (yellow arrowhead) strongly expressing MUC21D, which was a mixture of solid and micropapillary elements, filled a lymphatic canal. Magnification of all images is 200×.

High expressors of MUC21D and MUC21P were significantly related to lymphatic canal invasion but not vascular invasion (Fig 5A and 5B) (Table 2, MUC21P, 22/79 (27.9%), vs. 1/37 (2.7%), Pearson x2 test, *P* = 0.0009; MUC21D, 12/29 (41.4%), vs. 11/87 (12.6%), *P* = 0.0021). On the other hand, high expressors of MUC21C showed no significant relationship to vessel invasions. Additionally, high expressors of MUC21D showed significantly higher frequency of lymph node metastasis and advanced stage tumors (Table 2). These results supported the notion that frequent lymphatic canal invasion is a biological basis for the aggressiveness of mPAP elements [1, 8–11].

**Table 2:** Correlation between MUC21 expression and pathological factors in *EGFR*-mutated lung adenocarcinomas.

	MUC21P low expressor (N = 37)	MUC21P high expressor (N = 79)		MUC21C low expressor (N = 70)	MUC21C high expressor (N = 46)		MUC21D low expressor (N = 87)	MUC21D high expressor (N = 29)	
	% (N)	% (N)	*P*	% (N)	% (N)	*P*	% (N)	% (N)	*P*
**Size **			0.8242			0.8340			1.0000
≤ 20 mm	29.7(11)	26.6(21)		28.6(20)	26.1(12)		27.6(24)	27.6(8)	
> 20 mm	70.3(26)	73.4(58)		71.4(50)	73.9(34)		72.4(63)	72.4(21)	
Mean (range)	26.3(6–50)	25.7(8–53)		26.5(6–53)	25.0(8–46)		25.3(6–50)	25.2(12–46)	
**Vascular invasion **			0.3894			0.3075			0.6493
Present	24.3(9)	34.2(27)		27.1(19)	37.0(17)		29.9(26)	34.5(10)	
Absent	75.7(28)	65.8(52)		72.9(51)	63.0(29)		70.1(61)	65.5(19)	
**Lymphatic invasion**			0.0009*			0.2338			0.0021*
Present	2.7(1)	27.9(22)		15.7(11)	26.1(12)		12.6(11)	41.4(12)	
Absent	97.3(36)	72.1(57)		84.3(59)	73.9(34)		87.4(76)	58.6(17)	
**Lymph node metastasis**			0.4473			0.8074			0.0125*
No metastasis	86.5(32)	75.8(47)		82.9(58)	80.4(37)		87.4(76)	65.5(19)	
Metastatic	13.5(5)	20.2(16)		17.1(12)	19.6(9)		12.6(11)	34.5(10)	
**Stage**			0.1339			0.8124			0.0314*
I	89.2(33)	76.0(60)		81.4(57)	78.3(36)		85.1(74)	65.5(19)	
II,III, IV	10.8(4)	24.0(19)		18.6(13)	21.7(10)		14.9(13)	34.5(10)	

High expressor was defined as a tumor with a score more than or equal to 10 (MUC21P), 70 (MUC21C), 5 (MUC21D); Low expressor was defined as a tumor with a score less than 10 (MUC21P), 70 (MUC21C), 5 (MUC21D); N, number; *P* values were calculated using the Fisher’s exact test; Asterisk (*), statistically significant

### MUC21D levels and types of *EGFR* mutations

No significant difference in the types of *EGFR* mutations (major (exon 19, 21) or minor (exon 18, 20) mutations) between MUC21D high expressors and MUC21D low expressors was found (major mutation frequency, 21/22 (95.5%) vs 61/68 (89.7%), Fisher’s exact test, *P* = 0.6738).

### Relationship between MUC21D levels and postoperative outcome of *EGFR*-mutated LADCs

**Fig 6:**
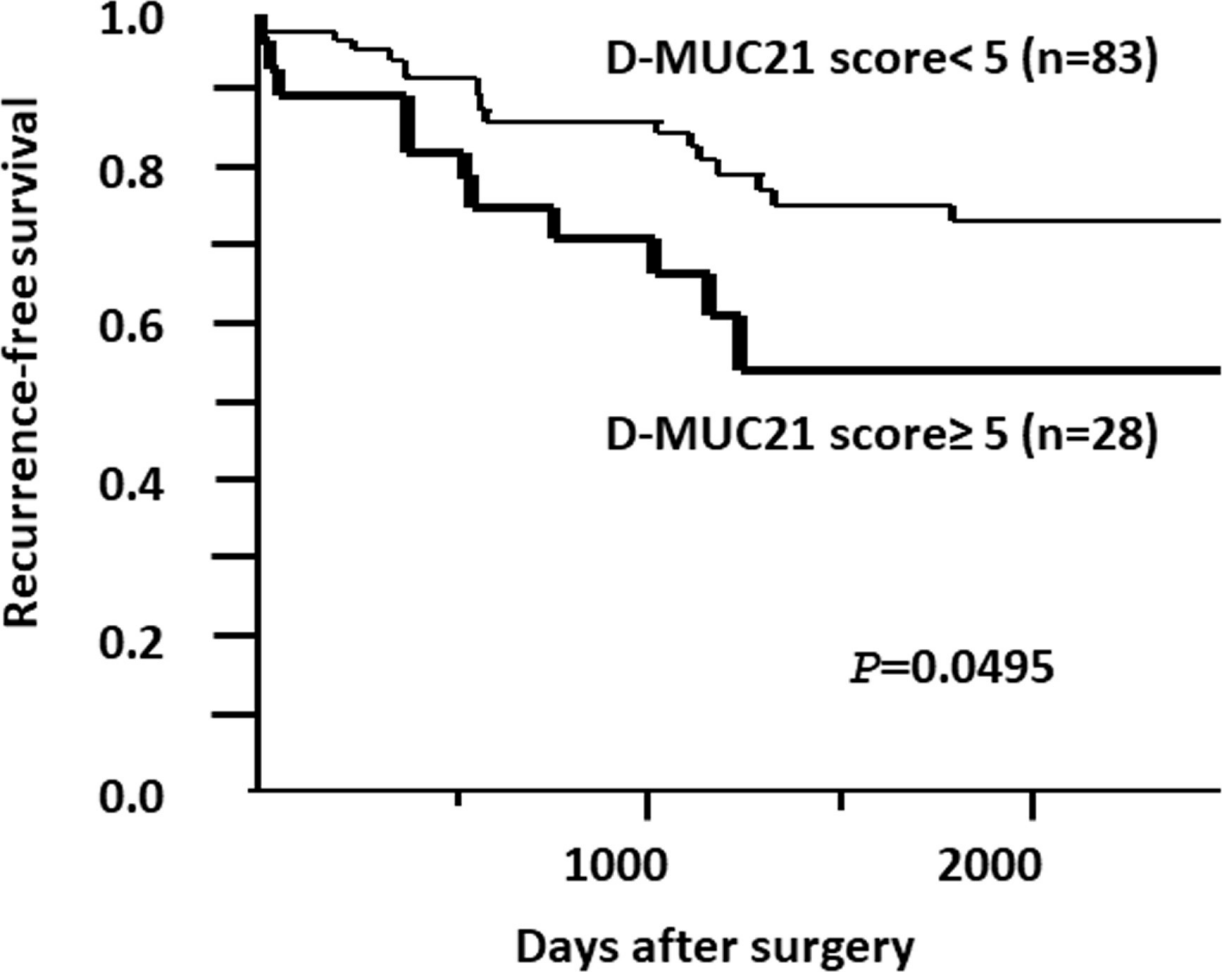
Kaplan-Meier recurrence-free survival (*RFS*) curves for the association between the level of MUC21D and disease recurrence. The *RFS* was significantly worse in MUC21D high expressors. A *P*-value was calculated using the log-rank test. n, number of tumors examined. A total of 111 patients with *EGFR*-mutated lung adenocarcinomas were examined with a median follow-up period of 52 months (range: 4–155 months).

High expressors of MUC21D showed significantly worse *RFS* (5-year Recurrence-free survival 50.7% vs. 73.8%, log-rank test *P* = 0.0495) (Fig 6). High expressors of MUC21P and MUC21C showed slightly worse *RFS*, but no significant difference was found (MUC21P; 5-year *RFS* rate 60.4% vs. 84.2%, *P* = 0.0681 in the log-rank test, MUC21C; 5-year *RFS* rate 59.9% vs. 73.3%, *P* = 0.4867 in the log-rank test). Thus, MUC21D may be a useful prognostic marker for predicting the recurrence of *EGFR*-mutated LADCs.

### Relationship among MUC21D levels, mPAP elements, and postoperative outcome of *EGFR*-wild-type LADCs

For *EGFR*-wild type LADCs, MUC21D high expressors had a significantly greater proportion of mPAP elements (Mann-Whitney test *P* = 0.0107). In addition, patients with MUC21D high expressors had slightly worse *RFS*, but no significant difference was found (5-year *RFS* rate 38.6% vs. 73.7%, *P* = 0.1958 by a log-rank test).

## Discussion

The most interesting finding was that the three varieties of MUC21 immunohistochemical expressions were related to different histological elements in *EGFR*-mutated LADCs. MUC21P and MUC21C were related to both LEPL and mPAP elements, and MUC21D was exclusively related to mPAP elements. The mPAP element is generated from a lepidic element through an intermediate LEPL element [7]. Thus, MUC21 protein with a specific glycosylation status may be involved in the progression of *EGFR*-mutated LADCs, particularly at the stage where tumors are transforming from lepidic to mPAP through LEPL lesions. In particular, MUC21D may be an important factor to confer aggressiveness to mPAP because MUC21D high expressors had frequent lymphatic canal invasion and a high recurrence rate.

Reviewing the results shown above, you may notice one discrepancy that a significant association between MUC21 expression and mPAP-predominant subtypes was failed to be found. We consider that it could be a limitation that sample number of mPAP-predominant LADCs in our series was too small to produce a statistical difference.

Yi et al. revealed that mouse Muc21/epiglycanin prevented cell-extracellular matrix interactions and interfered with intercellular adhesions, which suggests that Muc21/epiglycanin blocks surface integrins and intercellular adhesion molecules [12, 13]. The mPAP element of LADCs reduces the levels of adhesion molecules [11]. Miyoshi et al. reported that the mPAP element loses integrin-mediated adhesion to the basal membrane [14], which may be how MUC21 produces mPAP morphologies. Moreover, muc21/epiglycanin interferes with the immune response [5], which may promote the aggressiveness of mPAP elements.

MUC21D was associated with aggressive behavior of *EGFR*-mutated LADCs. The mAb heM21D binds to both the unmodified core polypeptide of MUC21 and Tn-MUC21, and mAb heM21C binds to Tn-MUC21, T-MUC21, and sialyl T-MUC21 [3]. The glycoproteins of tumor cells are often abnormal in structure. In particular, mucin-type O-glycans have several cancer-associated structures, including the T, Tn, and sialyl-Tn antigens, because of an elongation defect during glycosylation [15]. It is suggested that upon the progression of *EGFR*-mutated LADCs, particularly at the stage where tumors are transforming from pure lepidic to mPAP through LEPL lesions, biosynthesis of O-glycans on MUC21 is halted and MUC21 with less glycosylated and/or truncated glycans is formed. These structural changes can alter the cellular functions, such as adhesive properties, and the potential to invade and metastasize. Among some cancer-associated glycosylations, the Tn antigen is a marker for poorly differentiated colon adenocarcinomas, and an increased occurrence is associated with advanced cancer, invasive and highly proliferative tumors, metastasis, and poor outcomes [16]. These observations supported the idea that MUC21D, which has shorter glycosylated sugar chains (like Tn-MUC21), forms highly malignant histological components such as mPAP elements.

On the other hand, among *EGFR*-wild type LADCs, MUC21D expression was related to mPAP elements and poor outcomes. These results further supported the former idea that MUC21D produces mPAP features and their aggressive behavior.

In conclusion, we demonstrated a relationship between MUC21 expression and the progression of *EGFR*-mutated LADCs. MUC21 may have clinical utility as a prognostic marker for predicting the recurrence of *EGFR*-mutated LADCs and may be a target of molecular therapy. However, the molecular function of MUC21 remains largely unknown, and further studies are needed.

## Supporting information

S1 Table The minimal underlying data set from *EGFR*-mutated lung adenocarcinomas. (XLSX)
